# Early maternal care may counteract familial liability for psychopathology in the reward circuitry

**DOI:** 10.1093/scan/nsy087

**Published:** 2018-09-25

**Authors:** Nathalie E Holz, Regina Boecker-Schlier, Christine Jennen-Steinmetz, Erika Hohm, Arlette F Buchmann, Dorothea Blomeyer, Sarah Baumeister, Michael M Plichta, Günter Esser, Martin Schmidt, Andreas Meyer-Lindenberg, Tobias Banaschewski, Daniel Brandeis, Manfred Laucht

**Affiliations:** 1 Department of Child and Adolescent Psychiatry and Psychotherapy, Central Institute of Mental Health, Medical Faculty Mannheim / Heidelberg University, J5, Mannheim, Germany; 2 Department of Biostatistics, Central Institute of Mental Health, Medical Faculty Mannheim, Heidelberg University, J5, Mannheim, Germany; 3 University Outpatient Clinic of the Institute for Psychiatric and Psychosomatic Psychotherapy, Central Institute of Mental Health, Medical Faculty Mannheim / Heidelberg University, J5, Mannheim, Germany; 4 Department of Psychiatry and Psychotherapy, Central Institute of Mental Health, Medical Faculty Mannheim / Heidelberg University, J5, Mannheim, Germany; 5 Department of Psychiatry, Psychosomatic Medicine and Psychotherapy, Goethe-Universität Frankfurt am Main,Hoffmann-Str. 10, Frankfurt am Main, Germany; 6 Department of Psychology, University of Potsdam, Karl-Liebknecht-Str. 24-25. OT Golm, Potsdam, Germany; 7 Department of Child and Adolescent Psychiatry, University of Zurich, Neumünsterallee 9, Zurich, Switzerland; 8 Center for Integrative Human Physiology, University of Zurich, Winterthurerstr. 190, Zurich, Switzerland; 9 Neuroscience Center Zurich, University of Zurich and ETH Zurich,Winterthurerstrasse 190, Zurich, Switzerland

**Keywords:** maternal care, ADHD, ventral striatum, fMRI, resilience, aggression

## Abstract

Reward processing is altered in various psychopathologies and has been shown to be susceptible to genetic and environmental influences. Here, we examined whether maternal care may buffer familial risk for psychiatric disorders in terms of reward processing. Functional magnetic resonance imaging during a monetary incentive delay task was acquired in participants of an epidemiological cohort study followed since birth (*N* = 172, 25 years). Early maternal stimulation was assessed during a standardized nursing/playing setting at the age of 3 months. Parental psychiatric disorders (familial risk) during childhood and the participants’ previous psychopathology were assessed by diagnostic interview. With high familial risk, higher maternal stimulation was related to increasing activation in the caudate head, the supplementary motor area, the cingulum and the middle frontal gyrus during reward anticipation, with the opposite pattern found in individuals with no familial risk. In contrast, higher maternal stimulation was associated with decreasing caudate head activity during reward delivery and reduced levels of attention deficit hyperactivity disorder (ADHD) in the high-risk group. Decreased caudate head activity during reward anticipation and increased activity during delivery were linked to ADHD. These findings provide evidence of a long-term association of early maternal stimulation on both adult neurobiological systems of reward underlying externalizing behavior and ADHD during development.

## Introduction

Reward processing is one of the key neuronal phenotypes altered in externalizing (Finger *et al.*, 2011; Kappel *et al.*, 2015; von Rhein *et al.*, 2015) and in internalizing disorders (Stringaris *et al.*, 2015). One of the major components of the reward circuitry is the striatum, including the caudate head, and its ventral part is typically involved in evaluating reward value and predicting reward *vs* risk (Haber, 2011). Most prominently, altered striatal activity has been shown in attention deficit hyperactivity disorder (ADHD). Several lines of research have confirmed the importance of this intermediate phenotype, revealing a differential striatal activity pattern depending on the phase of reward processing, with hypoactivity during reward anticipation and hyperactivity during delivery (Plichta *et al.*, 2009; Furukawa *et al.*, 2014; Plichta & Scheres, 2014; Kappel *et al.*, 2015; von Rhein *et al.*, 2015). Although a high heritability of ADHD has been reported (Chang *et al.*, 2013; Banaschewski *et al.*, 2017), studies have also highlighted that mother–child interactions might also play an important role (Pauli-Pott *et al.*, 2017).

Interestingly, ventral striatum (VS) activity has been shown to be susceptible to adverse (environmental) influences, which may affect the quality of parenting. Evidence highlighting the importance of early adversity for the neural circuitry of reward processing has mainly been provided during monetary incentive delay (MID) tasks (Knutson *et al.*, 2001; Kirsch *et al.*, 2003) (but see also Olino *et al.* (2014) for results with a reward guessing task), with a differential impact on the two phases of reward processing, i.e. anticipation and delivery. Most studies reported reduced activation in the basal ganglia during reward anticipation as a function of early adversity (Dillon *et al.*, 2009; Mehta *et al.*, 2010; Boecker *et al.*, 2014; Holz *et al.*, 2017) and, strikingly, of familial liability to a broad range of psychiatric disorders (Gotlib *et al.*, 2010; Andrews *et al.*, 2011; Grimm *et al.*, 2014; Olino *et al.*, 2014; Vink *et al.*, 2016). Notably, this hyposensitivity during anticipation was accompanied by hypersensitivity in the basal ganglia during reward delivery during adulthood (Boecker *et al.*, 2014). However, conflicting evidence for reward processing during adolescence has also been provided by linking low parental warmth to increased striatal activity during anticipation (Casement *et al.*, 2014).

Although this vulnerability perspective is undoubtedly relevant, it seems equally important to identify protective factors (Vidal-Ribas *et al.*, 2015; Hoffmann *et al.*, 2016). One such protective factor might be the quality of mother–child interaction, although its effect on the reward circuitry has rarely been investigated. As an example, maternal interpersonal affiliation has been related to increased VS activity in female offspring during reward anticipation, while the opposite pattern applied to males (Schneider *et al.*, 2012). Morgan *et al.* (2014) provided further evidence of an impact of maternal warmth on motivation-related striatum activity in boys from low-income families; in boys exposed to maternal depression, maternal warmth during adolescence was related to increased VS activity, while maternal warmth during early childhood was associated with decreased VS activity. In a similar vein, in children exposed to maternal depression, low maternal authoritative parenting predicted a blunted feedback-related negativity during the delivery phase (Kujawa *et al.*, 2015). In sum, research has indicated a persistent alteration of affective processing as a function of early mother–infant interaction (Moutsiana *et al.*, 2014), specifically with regard to VS responding during reward processing (Schneider *et al.*, 2012; Morgan *et al.*, 2014).

Therefore, in the current study, we examined whether a higher level of early mother–child interaction very early in life, as a favorable environment, may counteract early adversity (`familial liability’) for psychiatric disorders in terms of reward processing and psychopathology in the offspring. Given that ADHD has been linked to lower striatal activity during anticipation and higher activity during delivery, we hypothesize higher striatum activity during anticipation, lower activity during delivery and fewer ADHD diagnoses as a function of high early maternal care in the high-risk group. The study therefore extends the current literature by investigating the long-term association of an early protective factor not only on the reward system but also on a behavioral level, in an epidemiological cohort of young adults followed since birth. We investigated this hypothesis by examining the interaction between parental psychiatric disorders (high familial risk) and measures of early mother–child interaction with regard to (i) characteristics of the offspring’s reward processing and to (ii) psychopathology during development in the offspring.

## Materials and methods

### Sample

**Fig. 1:**
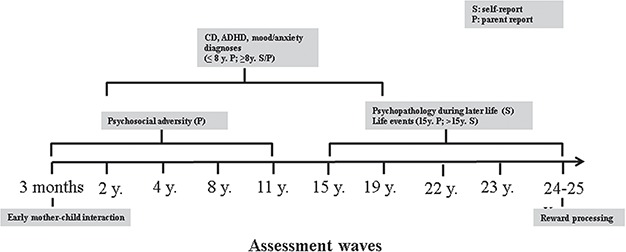
Assessment waves.

This investigation was conducted in the framework of the Mannheim Study of Children at Risk, an ongoing epidemiological cohort study of the long-term outcome of early risk factors (Laucht *et al.*, 2000). Of 309 participants (80% of the original sample), only currently healthy participants were included in the neuroimaging sample to avoid confounding by current impairment. The final sample for this investigation was *N* = 172. Further details regarding the sample and attrition are given in the supplement. All relevant assessments for this investigation are depicted in Figure 1. The study was approved by the ethics committee of the University of Heidelberg and a written informed consent was obtained from all participants.

### Assessments

#### Familial risk (parental psychiatric diagnoses)

The presence of current psychiatric diagnoses (disorders of adult personality and behavior F60-F69; mood (affective) disorders F30-F39, mental and behavioral disorders due to psychoactive substance use F10-F19; anxiety, dissociative, stress-related, somatoform and other non-psychotic mental disorders F40-F48) in biological parents until the participants’ age of 11 years was assessed using diagnostic interviews with the parents [Mannheim Parent Interview (MPI); Esser *et al.*, 1989)]. The MPI is a highly structured interview adapted from Rutter’s parent interviews (Cox and Rutter, 1985). It was conducted by informed trained psychologists at each of the five assessments during childhood (Figure 1), yielding a dichotomous variable (0 = not present, 1 = present). Further details are given in the supplement.

#### Early mother–child interaction

Videotapes of a 10 min standardized nursing and playing situation between mothers and their 3-month-old babies at our lab were recorded and evaluated by trained raters (κ > 0.83) using a modified version of the categorical system for microanalysis of the early mother–child interaction (Jörg *et al.*, 1994). Raters were blind to parental and child risk status. Nine measures of mother–infant interaction behavior were formed by coding a behavior as present or absent in a total of 120 5s intervals. Maternal stimulation included all attempts to attract the infant’s attention or to establish contact with him/her and was coded when the baby was gazing at the mother or the behaviors were clearly directed to the child. Eliciting behaviors can be vocal, facial or motor stimulation. As stimulation is positively coded, all eliciting behaviors must be appropriate to the infant’s state and communicative availability. Accordingly, stimulation reflects a maternal capacity to responsively motivate/stimulate her baby to interact with her. Maternal responsiveness comprised all behaviors executed in response to the infant behaviors (vocal, facial or motor). Additionally, infant vocal, facial and motor responsiveness was assessed accordingly to adjust maternal interaction behavior to the infant’s behavior. To compensate for differences in the mean between the three communication channels and to weight the channels equally, scores of vocal, facial and motor responsiveness and stimulation, respectively, were *z*-transformed and summarized to provide total scores of maternal stimulation, maternal responsiveness and infant responsiveness. Infant responsiveness was assessed accordingly and added as a covariate in all interaction models including maternal stimulation and responsiveness to ensure that the effects were specifically attributable to maternal behavior. The validity of the early interaction paradigm and the measures derived has been demonstrated in several publications (e.g. Laucht *et al.*, 2001; Buchmann *et al.*, 2010; Schmid *et al.*, 2011).

#### Environment

Psychosocial adversity was assessed until the age of 11 years and included information on adverse characteristics of the parents, their partnership and the family environment. Likewise, exposure to life stress (15–25 years) was assessed by a semi-structured parent interview. More information on these measures is provided in the supplement.

#### Child and adolescent psychopathology

Sum scores for the presence of ADHD diagnoses, disruptive symptoms/conduct disorder (CD) diagnoses and mood/anxiety diagnoses during childhood and adolescence were assessed using diagnostic interviews with the parents (MPI; Esser *et al.*, 1989) until age 11 years and with the children at ages 8 and 11 years. At age 15 years, the Schedule for Affective Disorders and Schizophrenia for School-Age Children (K-SADS-PL; Delmo *et al.*, 2000) was conducted independently with parents and adolescents and, at the age of 19 years, the Structured Clinical Interview for DSM-IV (Wittchen *et al.*, 1997) was performed with the offspring. A diagnosis was defined as present when criteria were met in either the parent or adolescent interview. The presence of a diagnosis (0 = not present, 1 = present) for each assessment (*N* = 6) was then added up to a sum score. Additionally, a global score of psychopathology was calculated by summing up all diagnosis-specific sum scores.

#### Psychopathology during early adulthood

To evaluate behavior problems in adolescence and adulthood (15–25 years), the participants completed the Youth Self-Report (YSR; Achenbach, 1991a) and the Young Adult Self-Report (YASR; Achenbach, 1991b), respectively. We focused on the subscales `externalizing behavior’ and `aggressive behavior’. Scores (available for *N* = 169) were *z*-standardized to form a composite sum score of the five assessments.

### Reward task

**Fig. 2:**
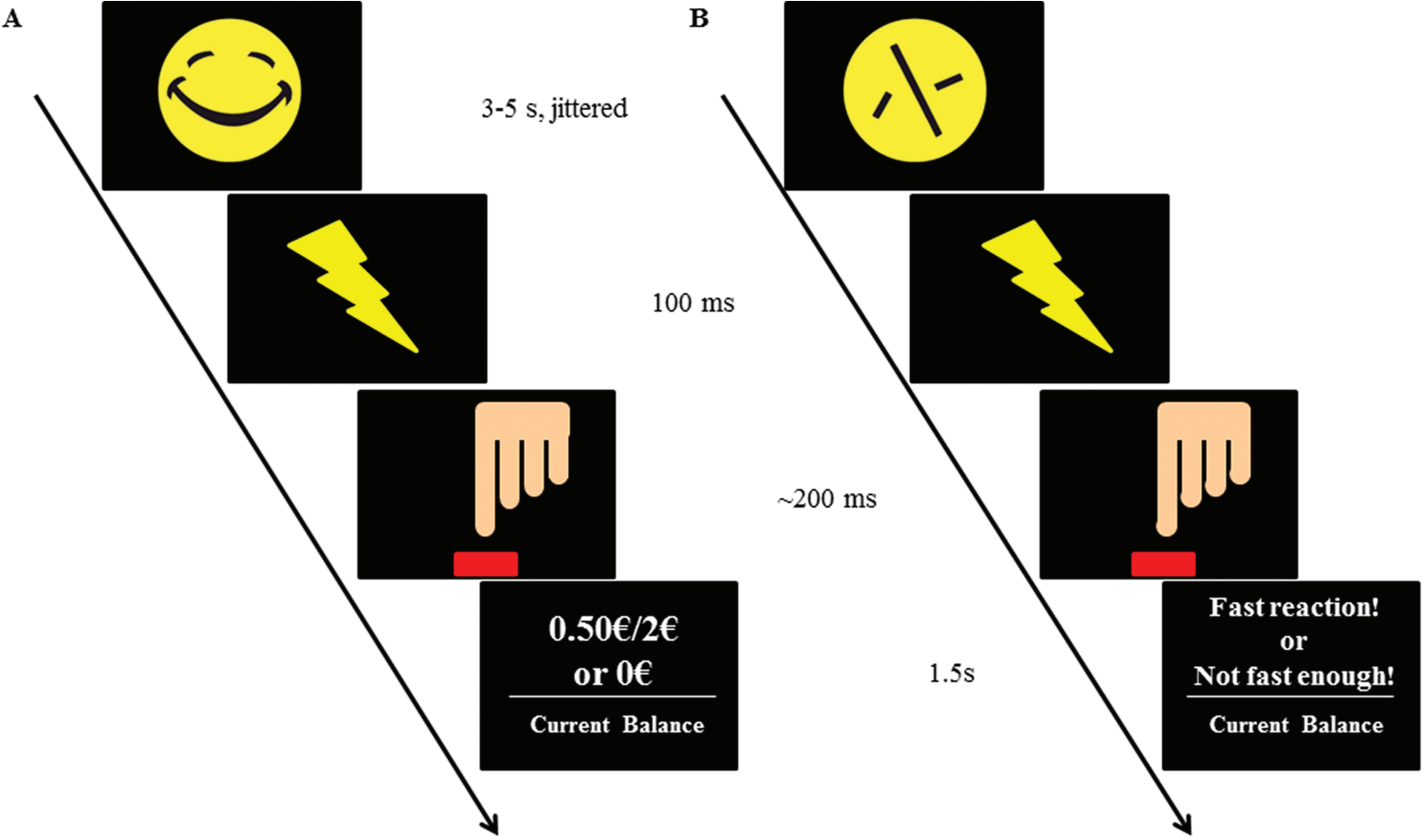
Reward paradigm. (A) Monetary trial. (B) Verbal trial.

The reward paradigm used in this study was a modified version of the MID task (Kirsch *et al.*, 2003), which separates reward anticipation (3–5s jittered) and delivery (1.5s), and yields reliable and robust activation of the VS (Boecker *et al.*, 2014; Holz *et al.*, 2017). The task does not include a neutral condition and requires a button press directly after a flash target following a cue indicating the type of reward. Targets followed either a laughing or a scrambled smiley, signaling either monetary feedback (0 or 0.50 or 2€ for boost trials) or the control condition, in which only verbal feedback was given (Figure 2; details in the supplement).

### Functional magnetic resonance imaging (fMRI) parameters and data analysis

Functional and structural magnetic resonance imaging (MRI) was performed at the age of 25 years using a 3 Tesla scanner (Magnetom TRIO, Siemens, Erlangen, Germany) with a standard 12-channel head coil. For functional imaging, a total of 400 volumes with 36 slices (matrix 64 × 64, resolution 3.43 × 3.43 × 3 mm, repetition time = 2210 ms, echo time = 28 ms, flip angle = 90°) covering the whole brain were acquired. Additionally, 1 × 1 × 1 mm T1-weighted anatomical images with 192 slices covering the whole brain (matrix 256 × 256, repetition time = 2300 ms, echo time = 3.03 ms, 50% distance factor, field of view 256 × 256 × 192 mm, flip angle 9°) were acquired. Functional images were analyzed using statistical parametric mapping (SPM8, http://www.fil.ion.ucl.ac.uk/spm) implemented in Matlab 7.12. (Mathworks Inc., Natick, MA, USA) with standard preprocessing steps, as depicted in the supplement.

First-level contrast images reflecting activation to (i) the anticipation of monetary *vs* verbal trials (cue onset) and (ii) the delivery contrast of win *vs* no-win trials (pooled over monetary and verbal feedback) were used. General task effects were obtained using whole-brain family-wise error (FWE) correction at *P* < 0.05. These contrasts were entered into second-level group multiple regression analyses (separate regressions for anticipation and delivery), with the interaction term between parental psychiatric diagnoses and maternal stimulation or maternal or infant responsiveness, respectively, as the main predictor while all main effects and sex were entered as additional covariates. Infant responsiveness was additionally controlled for in the interaction models including maternal measures, i.e. maternal stimulation by familial risk and maternal responsiveness by familial risk, to ensure that the effects could be solely attributed to maternal behavior. However, the results did not differ when this additional covariate was not included. In a first step, we used a hypothesis-driven region of interest (ROI) approach. As the striatum is a crucial region during reward processing, with the caudate head explicitly highlighted as being functionally compromised in ADHD (Plichta and Scheres, 2014) and CD (Blair *et al.*, 2016; Holz *et al.*, 2017), this region was defined as ROI, using an anatomical mask comprising 209 voxels for the right and 216 voxels for the left caudate head, implemented in the Wake Forest University PickAtlas v2.4 (Maldjian *et al.*, 2003), where a *P* < 0.05 FWE correction was applied. In a second step, exploratory whole-brain analyses were conducted at *P* < 0.05 FWE corrected. Tables depicting whole-brain activation at an uncorrected threshold of *P* = 0.001 are in the supplement. All corrections were performed on the voxel level and only fMRI results were FWE corrected. In the case of significant effects, mean contrast values of each participant were extracted from the cluster and exported to SPSS Statistics 20 (IBM, Armonk, NY), enabling visualization in scatterplots. As all plots were adjusted for covariates, negative values can emerge. Further, the association between the contrast values and psychopathology and the interaction effect of measures of mother–child interaction and familial risk on psychopathology were calculated using linear regression analyses in SPSS. Moreover, if not otherwise stated, all interactions were additionally investigated following the recommendation by Keller (2014), i.e. including all familial risk × covariate and maternal stimulation × covariate interactions. These results were similar to those of the original analyses and are depicted in the supplement. Regions of significance (RoS) were calculated using a web-based program freely available at http://www.yourpersonality.net/interaction (cf. Roisman *et al.*, 2012) (more detail in the supplement). A regression-based mediation model was tested using the PROCESS macro for SPSS (Hayes, 2013) to examine the indirect effect with caudate activation as a mediator of the relationship between maternal stimulation and ADHD using a bootstrap estimation approach with 10 000 samples [95% confidence interval (CI)].

To better establish specificity of effects, we additionally controlled for several confounders in the separate secondary analyses for each covariate. First, as environmental adversity has previously been related to differential reward processing (Boecker *et al.*, 2014), we ensured that the effects cannot be explained by psychosocial adversity by controlling for this variable. Further, since family history of externalizing disorders and of affective disorder are risk variables of very different importance, particularly in terms of reward processing, we excluded parental mood disorder diagnoses in the familial risk score (which now only entailed disorders of adult personality and behavior F60-F69; mental and behavioral disorders due to psychoactive substance use F10-F19; anxiety, dissociative, stress-related, somatoform and other non-psychotic mental disorders F40-F48) in a secondary analysis to demonstrate that the impact of familial risk cannot be attributed to the familial risk for affective disorders. Likewise, we further controlled for lifetime internalizing diagnoses in the offspring. Moreover, as the mother participated in the parent–child interaction, which should mainly be influenced by the mother's psychiatric history, the interaction patterns were calculated separately for maternal or paternal psychopathology by maternal stimulation.

## Results

### Sample characteristics

Individuals with high familial risk received lower maternal stimulation and had more psychopathology during the lifetime, such as ADHD, CD, mood and anxiety diagnoses and aggression as well as externalizing behavior during later life (Table 1). Moreover, higher maternal stimulation predicted a decreased level of ADHD (β = −0.18, *P* = 0.008) but was unrelated to other psychiatric disorders. There were no further significant associations with the main predictors.

**Table 1:** Sample characteristics by presence of parental psychiatric disorder during childhood

Parental psychiatric diagnosis	Not present	Present	Test statistics	P-value
N (%)	89 (51.7)	83 (48.3)		
Males, N (%)	35 (39.33)	37 (44.58)	X^2^(1) = 0.49	0.48
Maternal stimulation, mean (SD)^a^	0.23 (0.95)	−0.24 (1.00)	T(170) = 3.17	0.002
Maternal responsiveness, mean (SD)^a^	0.06 (0.98)	0.08 (1.01)	T(170) = −0.90	0.37
Infant responsiveness, mean (SD)^a^	−0.04 (0.94)	0.06 (1.05)	T(170) = −0.68	0.50
Sum of ADHD diagnoses, mean (SD)	0.27 (0.62)	0.58 (1.11)	T(170) = −2.24	0.03
Sum of disruptive behaviors and CD diagnoses, mean (SD)	0.15 (0.47)	0.36 (.85)	T(170) = −2.86	0.005
Aggression during later life, mean (SD)^a^	−1.21 (2.86)	−0.17 (3.39)	T(170) = −2.16	0.03
Externalizing behavior during later life, mean (SD)^a^	−1.49 (2.64)	−0.47 (3.27)	T(170) = −2.23	0.03
Sum of mood and anxiety disorder, mean (SD)	0.27 (0.58)	0.54 (1.03)	T(170) = −2.12	0.04
Psychopathology during lifetime, mean (SD)	0.63 (1.08)	1.33 (1.86)	T(170) = −2.98	0.003
Years in school, mean (SD)	11.85 (1.53)	11.55 (1.62)	T(170) = 1.24	0.22

Note: ^a^*z*-transformed scores

### Reward task

#### Task effects

Robust activations in the striatum were obtained during reward anticipation and delivery (see supplement).

#### Main effects

During reward anticipation, operationalized by the contrast of monetary *vs* verbal cues, high familial risk was associated with decreased activity in the offspring’s caudate head [left: t(169) = 3.99, p_FWE_ = 0.001; right: t(169) = 3.80, p_FWE_ = 0.003; see Supplementary Table S1]. During reward delivery, the opposite was found, indicating increased activity in the caudate head [left: *t*(169) = 3.22, p_FWE_ = 0.02; see Supplementary Table S2]. No effects were found for any measures of early mother–child interaction (p_FWE_ > 0.41; p_uncorr_ > 0.001).

#### Interaction effects

**Fig. 3:**
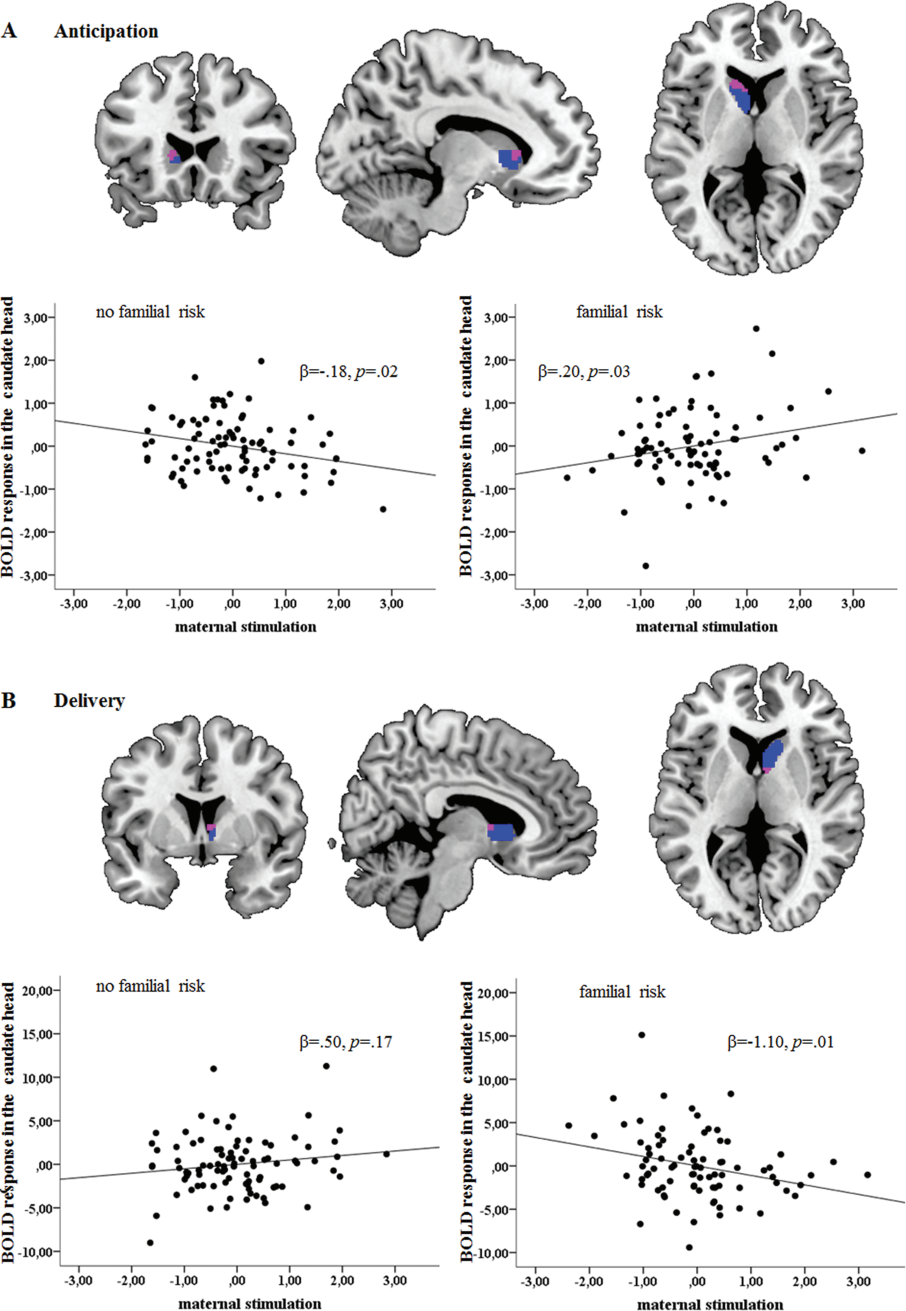
(A) Maternal stimulation × familial risk interaction effect on caudate head activity during reward anticipation (peak MNI −12 22 6). (B) Maternal stimulation × familial risk interaction effect on caudate head activity during reward delivery (peak MNI 6 4 6). Overlay (violet) of the cluster, in which the interaction effect was found and task-related caudate activation (blue). Effects are displayed at pFWE < 0.05 ROI corrected.

During reward anticipation*,* an interaction effect of familial risk with maternal stimulation on caudate head activity emerged [*t*(166)=3.63, p_FWE_=0.005; Figure 3A]. In detail, higher maternal stimulation was associated with increasing activity in the caudate head in individuals with high familial risk, while the reverse pattern was observed in no-risk participants. The analysis of RoS demonstrated significant differences between the two groups defined by the presence or absence of a familial risk at low levels and at very high levels of maternal stimulation (Figure S1 in the supplement). Further, on a whole-brain FWE-corrected level, significant interactions following the same pattern were obtained in regions of the extended reward system such as the supplementary motor area (Supplementary Figure S2), the cingulum and the middle frontal gyrus (see Supplementary Table S3 in the supplement for uncorrected whole-brain results).

Moreover, regarding maternal or infant responsiveness, all interactions failed to reach FWE significance (all p_FWE_’s > 11, see Supplementary Tables S4 and 5 for uncorrected results) on a whole-brain level and in the caudate.

In analogy to the already described opposing main effects during the two reward processing phases, the interaction followed the opposite direction during reward delivery in the right caudate [*t*(166) = 2.89, p_FWE_ = 0.04; Figure 3B; see Supplementary Table S6 for uncorrected whole-brain results], revealing a significant decrease in caudate activity with increasing maternal stimulation in individuals with high familial risk (β = −1.10, *P* = 0.01). Low levels and very high levels of maternal stimulation have driven the effect (Supplementary Figure S1). A break-down of the delivery contrast and all analyses including covariate by predictor interactions are depicted in the supplement.

Regarding maternal or infant responsiveness, all interactions failed to reach FWE significance (all p_FWE_’s > 0.13, see Supplementary Tables S7 and 8 for uncorrected results) on a whole-brain level and in the caudate.

### Interaction effects on child and adolescent psychopathology

**Fig. 4:**
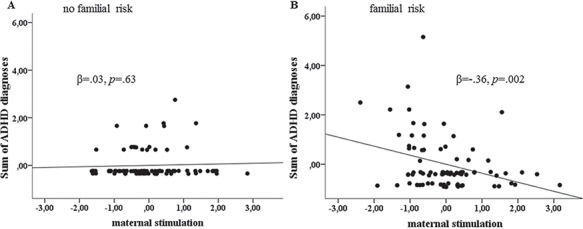
Maternal stimulation × familial risk interaction effect on ADHD diagnoses. (A) Maternal stimulation had no effect on ADHD in the offspring with low familial risk. (B) Higher maternal stimulation was associated with decreasing ADHD diagnoses in participants with high familial risk.

Familial risk and maternal stimulation interacted to predict general psychopathology in the offspring (β = −0.52, *P* = 0.03), with the association between maternal stimulation and the offspring’s psychopathology only being significant in those with high familial risk (β = −0.53, *P* = 0.007). When specifically investigating this interaction pattern with respect to ADHD, an interaction effect between familial risk and maternal stimulation on ADHD diagnoses emerged (β = −0.38, *P* = 0.005; Figure 4). Specifically, in the offspring with high familial risk, higher maternal stimulation was associated with fewer ADHD diagnoses (β =  −0.36, *P* = 0.002), while no such pattern emerged in the low-risk group. Similar to the RoS analyses presented above, low levels and very high levels of maternal stimulation had a detrimental or beneficial effect, respectively, on ADHD in individuals with high familial risk (Supplementary Figure S3). With regard to CD (*P* = 0.12) and internalizing disorders, the interaction failed to reach significance (*P* = 0.80). Likewise, the interactions between familial risk and maternal or infant responsiveness affected neither ADHD (*P*’s > 0.17) nor disruptive behaviors/CD (*P*’s > 0.78). All results remained unchanged when child and adolescent psychopathology was considered between 4 and 19 years of age only. Similar to the observed pattern for child and adolescent psychopathology, the effects persisted regarding externalizing symptoms during early adulthood (see supplement).

### Sensitivity analyses

An additional control for psychosocial adversity and life events did not change the results.

Likewise, the results remained significant after controlling for lifetime internalizing psychopathology in the offspring and when all covariate by predictor interactions were included. Further, the results cannot be attributed to familial risk for affective disorders.

Notably, the reward and ADHD results were specific for the interaction patterns between maternal psychopathology and maternal stimulation. All specificity analyses are described in more detail in the supplement.

### Association between activity in the caudate head and child and adolescent psychopathology

**Fig. 5:**
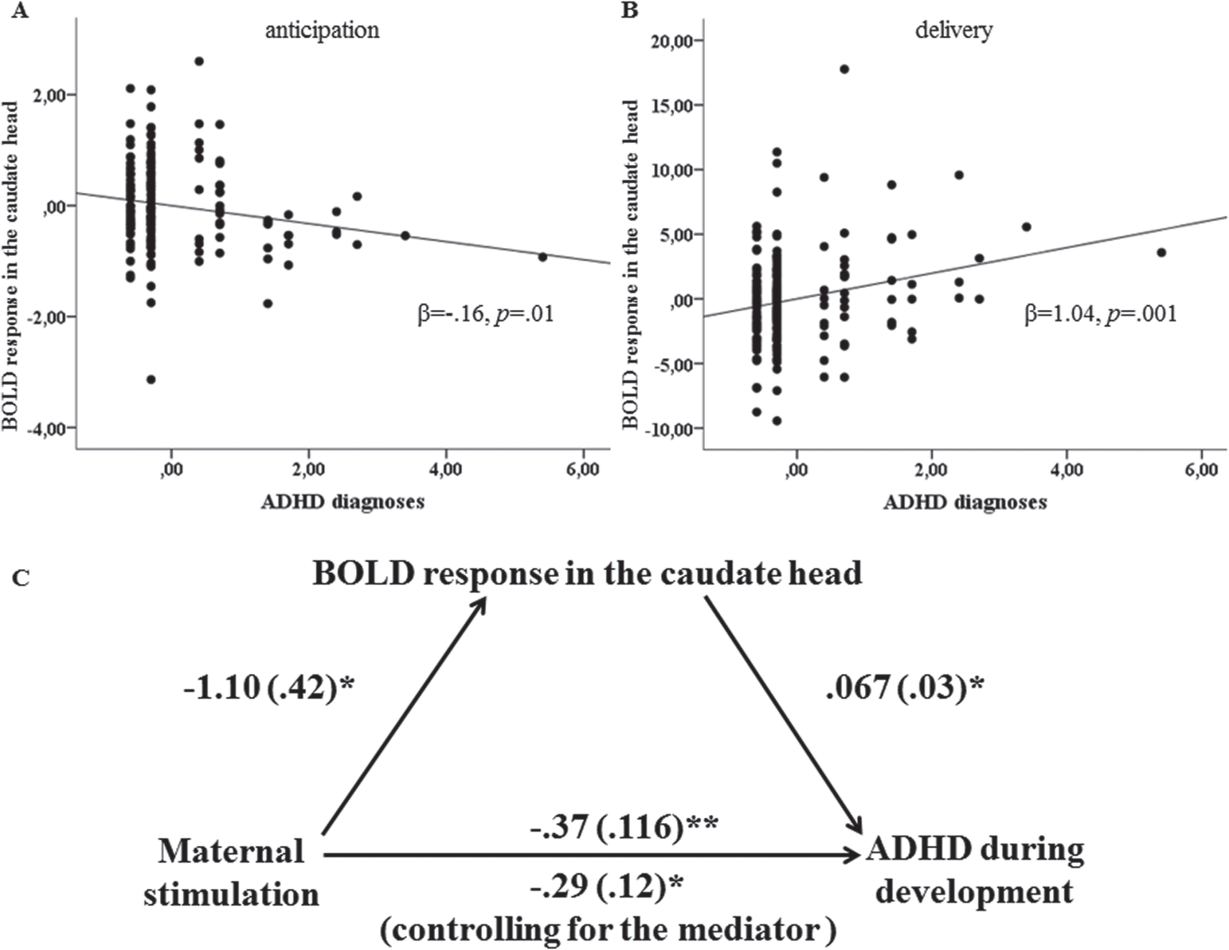
Association between ADHD diagnoses and activity in the caudate head during anticipation (A) and delivery (B). (C) Mediation analysis showing that caudate activity during the delivery phase partially mediated the association between maternal stimulation and ADHD.

Interestingly, opposing associations of caudate head activity during anticipation and delivery with ADHD were found (Figure 5A and B).

### Mediation analysis

Mediation analyses revealed that, in the high-risk group, the relationship between maternal stimulation and ADHD was partially mediated by caudate head activity during delivery (β = −0.0736; CI: −0.1790 – −0.0168, Figure 5C) but not during anticipation (β = −0.0339; CI: −0.1231 – −0.0009), while no such relationships emerged in the low-risk group.

## Discussion

The present prospective study over 25 years investigated whether early maternal care might counteract familial risk in terms of reward processing and externalizing disorders. Specifically, more maternal stimulation was related to more activation in the caudate head during reward anticipation in the offspring with high familial risk, while the opposite pattern emerged in the low-risk group. In contrast, more maternal stimulation was associated with less caudate head activity during reward delivery and with reduced levels of externalizing disorders during development in the high-risk group only. Remarkably, the results were independent of internalizing psychopathology, which has also been related to aberrant reward system functioning, and specifically related to maternal psychopathology. Moreover, caudate head activity was lower during reward anticipation and higher activity during delivery with higher levels of externalizing disorders. Caudate activity during delivery and, at a trend level, during anticipation mediated the relationship between maternal stimulation and ADHD in the high-risk group.

### The effect of familial risk on reward processing

To date, most studies investigated the detrimental effects of early environmental adversity (Dillon *et al.*, 2009; Mehta *et al.*, 2010; Boecker *et al.*, 2014) and familial liability (Gotlib *et al.*, 2010; Andrews *et al.*, 2011; Grimm *et al.*, 2014; Olino *et al.*, 2014; Vink *et al.*, 2016) on the brain’s reward circuitry, with blunted VS activity during reward anticipation as a common denominator. The present findings confirm that high familial risk is associated with hypoactivation during reward anticipation, but as part of a differential VS response profile including also hyperactivation during delivery. An altered sensitivity to reward cues in their environment may render individuals with an adversity background, such as familial risk, in particular need of stimulating care to increase the awareness of stimulus–reward contingencies.

### The protective effect of maternal stimulation on reward processing in the high risk group

Interestingly, our results show that a higher level of maternal stimulation may alter the offspring’s reward sensitivity depending on the presence of familial risk, thereby buffering the adverse effect of high familial risk.

Increased activity during reward anticipation but decreased activation with increasing levels of maternal stimulation during delivery in the high-risk group might indicate resilient functioning, which is supported by the fact that the participants were healthy at the time of the fMRI measurement. In fact, such deviations of caudate responding during reward anticipation and delivery in opposite directions have been observed in child (Scheres *et al.*, 2007; Furukawa *et al.*, 2014) (but see Paloyelis *et al.*, 2012; von Rhein *et al.*, 2015) and adult ADHD during anticipation (Strohle *et al.*, 2008; Plichta *et al.*, 2009; Hoogman *et al.*, 2011; Carmona *et al.*, 2012; Kappel *et al.*, 2015) (but see Stoy *et al.*, 2011) and during delivery (Paloyelis *et al.*, 2012; Furukawa *et al.*, 2014; von Rhein *et al.*, 2015) (as also indicated in our results in Figure 5). Thus, the inverse response profile in the high-risk group might suggest a possible protective role of these activation patterns against ADHD. Remarkably, the interaction effects appear to be specific to ADHD, as the results remained significant after controlling for internalizing psychopathology, which itself has been related to blunted VS responding (Hanson *et al.*, 2015; Stringaris *et al.*, 2015; Luking *et al.*, 2016). Generally, while the anticipation phase is more reflective of `wanting’ a reward, which addresses the motivation to receive an incentive, reward delivery mostly covers the `liking’ aspect, including the hedonic effect of the reward itself (Berridge *et al.*, 2009). Thus, higher caudate activation during anticipation with increasing maternal stimulation, as seen in the high familial risk group, might indicate an augmented salience of the monetary reinforcer, which in turn might enhance the ability of reward-predicting cues to elicit appropriate goal-directed and approach actions. In contrast, decreasing activation during delivery might suggest a lower hedonic-inducing effect.

In contrast, no such interaction effect was found regarding maternal responsiveness (or infant responsiveness). This is in accordance with previous results confirming the superiority of maternal stimulation over responsiveness across many facets, including the cortisol stress response (Schmid *et al.*, 2013). As such, this may indicate that behavior which actively elicits communication between the mother and the child might be more important for shaping the reward circuitry than passive responding.

Unexpectedly, increasing maternal stimulation was related to decreasing caudate head activity in the context of low familial risk for psychopathology (Figure 3A) during reward anticipation. Notably, this pattern was also seen in areas of the extended reward system such as the supplementary motor area (Supplementary Figure S2). While this seems counterintuitive at first glance, it is important to stress that stimulation was higher *per se* in the no familial risk group (Table 1). A plausible explanation is provided by Kochanska (1997), who hypothesized that, in cases of high quality of mutual interaction, maternal control and coercion should be decreased, as the child may be more receptive to parental goals. This might imply that the highest levels of stimulation might have an overstimulation/intrusiveness effect in the low-risk group. Indeed, the link between parental intrusiveness and psychopathology has already been discussed (Beebe & Steele, 2013), suggesting that stimulation in the midrange might be optimal for the low-risk individuals. However, it must be acknowledged that this potential explanation cannot be corroborated from our data since the quality of maternal interaction behavior was not assessed. Moreover, a visual inspection of the regression in the no risk group reveals that one participant had extreme levels of maternal stimulation, which might further indicate that the link between high stimulation and low caudate activity in the no risk group might be tentative. When excluding this participant, the overall interaction remains significant [t(165) = 3.20, pFWE = 0.02], but the regression in the low-risk group fails to reach significance (*P* = 0.07), possibly indicating a less robust effect in the low-risk group.

The results of this study complement the picture of previous research on this topic, while also providing some conflicting evidence. For example, caudate responding during reward in boys exposed to maternal depression was shown to differ depending on maternal warmth assessed during early childhood *vs* adolescence (Morgan *et al.*, 2014). However, their findings are only partly in line with our results. Specifically, they found that during anticipation, more maternal warmth during childhood was related to decreasing striatum activation in boys exposed to maternal depression, while the opposite, i.e. increasing caudate activity, was associated with greater maternal warmth during adolescence. Thus, while their results regarding maternal warmth during childhood were in contrast to our results, the same pattern was seen during adolescence for the association between maternal warmth and striatal activation in both studies. This inconsistency may be due to the nature of familial risk, i.e. maternal major depressive disorder, the methodology, i.e. condition of interest *vs* baseline, and the sample, i.e. socially disadvantaged boys, in the aforementioned study when compared to our study. However, the interaction pattern on caudate activity during delivery was in accordance with our findings, i.e. higher maternal warmth during childhood was related to less striatal activation in the risk group during the outcome phase. Analogous interaction patterns have also been shown regarding event-related potentials, with maternal positive parenting (high control, high warmth) predicting an increased feedback-related negativity during delivery in the children of mothers with a history of depression (Kujawa *et al.*, 2015). Our results extend these findings by demonstrating a long-term protective effect of early mother–child interaction with regard to reward processing in those with a general risk of psychopathology and by showing an additional interaction effect of familial risk and maternal stimulation on ADHD.

### The protective effect of maternal stimulation on ADHD in the high risk group

In addition to the associations with caudate head activity during reward processing, we found an interaction effect relating to the amount of lifetime ADHD diagnoses. While maternal stimulation was associated with less ADHD diagnoses during lifetime in the risk group, no such relationship was seen in the no-risk group. Interestingly, psychosocial adversity has been regarded as a correlated not yet proven risk factor for ADHD (Thapar *et al.*, 2013). Although poor parenting alone is unlikely to cause ADHD and might rather be considered in terms of reverse causation (Thapar and Cooper, 2016), negative early caregiving is indeed discussed as aggravating ADHD symptoms in the offspring. Hence, our results indicate that high familial risk is associated with increased ADHD in the offspring and, interestingly, high maternal stimulation might buffer against this association in the high-risk group.

### Limitations

Despite the notable strengths of our study, such as the prospective design allowing us to refer back to an observation of early mother–child interaction, and a well-characterized sample, the results should be considered in light of some limitations. First, maternal stimulation was assessed only in infancy. However, longitudinal studies have shown that the positive as well as negative maternal constellations are stable from birth to adolescence and are uniquely predictive of children’s social-emotional outcomes across childhood and into adult life (Feldman, 2010). Thus, it can be assumed that a mother who displays poor or intensive stimulation with her 3-month-old infant will continue to interact accordingly during the child’s later development. Second, parental mental health assessment was only conducted until the participant’s age of 11 years, which may have led to increased false negatives in the healthy parents group. Third, while data on maternal psychiatric disorders were consistently available for all assessments, there were several missing values for paternal psychopathology. Fourth, increased activation during anticipation and decreased activation during delivery are interpreted as beneficial effects, based on findings showing that the reverse patterns are associated with environmental risk (Boecker *et al.*, 2014), ADHD (Furukawa *et al.*, 2014; von Rhein *et al.*, 2015) and risk of other psychopathologies (Luking *et al.*, 2016). However, we cannot rule out that these deviations may also represent compensatory effects. To test for the latter, the inclusion of individuals with current ADHD would have been necessary. Fifth, we focused on maternal interactive behavior. While this may have been considered as more important than father–child interaction back in 1986, future studies should also focus on paternal interactive behavior given the increasing importance attributed to fathers in parenting. Sixth, as reward processing was only assessed during adulthood, we were unable to track the direct impact of maternal care on motivation processing and its link to psychopathology.

## Conclusion

The present findings suggest a continuous and long-term association of early maternal interaction behavior on the neural underpinnings of reward processing up to adulthood and the offspring’s mental health, particularly in individuals with a high familial risk for psychiatric disorders. Maternal stimulation may thus serve as a protective factor that might offset the risk conferred by familial risk. If this is the case, therapeutic interventions should focus on improving maternal care in early mother–child interactions, particularly in those with a familial risk for psychopathology.

## Supplementary Material

Supplementary Data
