# Supplementary material for: Early maternal care may counteract familial liability for psychopathology in the reward circuitry
Source: Soc Cogn Affect Neurosci. 2018 Sep 25;13(11):1191–201. doi: 10.1093/scan/nsy087 (PMC6234324; doi:10.1093/scan/nsy087)
Supplement: Supplementary Data [file nsy087_supp.doc]

# Title page

**Early maternal care may counteract familial liability for psychopathology in the reward circuitry**

Nathalie E. Holz^1^, Ph.D., Regina Boecker-Schlier^1^, Ph.D., Christine Jennen-Steinmetz^2^, Ph.D., Erika Hohm^1^, M.A., Arlette F. Buchmann^1,3^, Ph.D., Dorothea Blomeyer^1^, Ph.D., Sarah Baumeister^1^, Ph.D., Michael M. Plichta^1,4,5^, Ph.D., Günter Esser^6^, Ph.D., Martin Schmidt^1^, M.D., Ph.D., Andreas Meyer-Lindenberg^4^, M.D., Ph.D., Tobias Banaschewski^1^*, M.D., Ph.D., Daniel Brandeis^1, 7-9^*, Ph.D., Manfred Laucht^1,6^*, Ph.D.

*Equal contribution

Central Institute of Mental Health, Medical Faculty Mannheim / Heidelberg University, J5, 68159 Mannheim, Germany, ^1^Department of Child and Adolescent Psychiatry and Psychotherapy, ^2^Department of Biostatistics, ^3^University Outpatient Clinic of the Institute for Psychiatric and Psychosomatic Psychotherapy,  ^4^Department of Psychiatry and Psychotherapy;

^5^Department of Psychiatry, Psychosomatic Medicine and Psychotherapy, Goethe-Universität Frankfurt am Main, Hoffmann-Str. 10, 60528 Frankfurt am Main, Germany, ^6^Department of Psychology, University of Potsdam, Karl-Liebknecht-Str. 24-25, 14476 Potsdam OT Golm, Germany; ^7^Department of Child and Adolescent Psychiatry, University of Zurich, Neumünsterallee 9, 8032 Zurich, Switzerland, ^8^Center for Integrative Human Physiology, Winterthurerstrasse 190, 8057 Zurich, Switzerland, ^9^Neuroscience Center Zurich, University of Zurich and ETH Zurich, Winterthurerstrasse 190, 8057 Zurich, Switzerland

**Running title:** maternal care counteracting familial risk

# Supplemental Information

**Methods** Sample, Familial Risk, Early mother-child interaction, Reward task, fMRI parameters and data analysis

**Results** Task effects, Interaction effects, Interaction effects on child and adolescent psychopathology, Interaction effects on psychopathology during later life, Specificity of the effects

**References**

**Table S1.** Negative association with familial risk during reward anticipation at p=001 uncorrected.

**Table S2**. Negative association with familial risk during reward delivery threshold of p=.001 uncorrected.

**Table S3.** Interaction between familial risk and maternal stimulation with regard to reward anticipation at p=.001 uncorrected.

**Table S4.** Interaction between familial risk and child responsiveness with regard to reward anticipation at p=.001 uncorrected.

**Table S5.** Interaction between familial risk and maternal responsiveness with regard to reward anticipation at p=.001 uncorrected.

**Table S6.** Interaction between familial risk and maternal stimulation with regard to reward delivery at p=.001 uncorrected.

**Table S7.** Interaction between familial risk and infant responsiveness with regard to reward delivery at p=.001 uncorrected.

**Table S8.** Interaction between familial risk and maternal responsiveness with regard to reward delivery at p=.001 uncorrected.

**Figure S1.** Region of Significance analysis of the interaction between maternal stimulation and familial risk with regard to reward processing.

**Figure S2.** A. Maternal stimulation (MS) x familial risk interaction on supplementary motor area activity during reward anticipation.

**Figure S3.** Region of Significance analysis of the interaction between maternal stimulation and familial risk with regard to ADHD.

# Method

## Sample

The initial sample consisted of 384 children of predominantly (>99.0%) European descent, born between 1986 and 1988. Infants were recruited from two obstetric and six children’s hospitals in the Rhine-Neckar Region of Germany and were included consecutively into the sample according to a two-factorial design intended to enrich and to control the risk status of the sample . To control for confounding effects of family environment and infant medical status, only firstborn children with singleton births and German-speaking parents were enrolled. Assessments were conducted at the age of 3 months and at regular intervals throughout development. Functional and structural magnetic resonance imaging was introduced in the most recent assessment at age 25 years. From the initial sample of 384 participants, 18 (4.7%) were excluded due to severe disabilities, and 57 (14.8%) were dropouts, leaving a final sample of 309 for the 25-year assessment and 201 for the fMRI investigation. Exclusion criteria were the usual contraindications for MRI (such as heart pacemaker, neurological abnormalities, history of seizures, unconsciousness or head trauma), current psychiatric disorders as assessed by the Structured Clinical Interview for DSM-IV , and psychotropic medication. Data was analyzable for N=183 after exclusion due to movement >3mm and due to psychiatric and somatic diseases for N=18. From these, mother-child interaction data and familial risk was missing for 11 participants, leaving a final sample of N=172. In terms of attrition, the dropout sample had higher obstetric (t(380)=4.17, p<.001) and psychosocial risk (t(380)=3.18, p=.002), more parental psychiatric diagnoses (t(380)=3.00, p=.003) as well as a lower level of maternal responsiveness (t(366)=-2.67, p=.008), however, were not different with regard to maternal stimulation, sex and sum of ADHD diagnoses. A comparison between those who participated in psychiatric assessment of the last assessment wave (including those with current diagnoses) versus those selected for the fMRI measurement, showed a higher level of maternal reactivity (t(293)=-2.22, p=.03) and lower obstetric risk (t(305)=2.81, p=.007) in the fMRI sample. No differences emerged regarding sex, ADHD, maternal stimulation, psychosocial adversity and parental psychiatric diagnoses, therefore the fMRI sample is likely to be unbiased in terms of environmental hardship and most of the variables of interest of the current investigation.

## Familial risk

A substantial degree of genetic transmission of parent-child resemblance for externalizing disorders has been demonstrated , however, various other parental psychiatric disorders including mood and anxiety disorders , substance use disorders and personality disorders have also been related to child externalizing behavior. In addition, all disorder groups have been linked to altered reward processing . Therefore, psychiatric diagnoses in biological parents included different diagnostic categories, such as personality, affective, substance-related and addictive, somatoform and stress disorders (0=not present, 1=present). 6 participants were excluded due to missing information on the mental health of their biological parents. The numbers of missing values for paternal psychiatric disorder for the five assessment waves were 2, 2, 6, 9, 13; here only maternal mental health was considered.

## Early mother-child interaction

Early mother-child interaction data was missing for 5 subjects.

## Environment

Psychosocial adversity included Information on adverse characteristics of the parents (low educational level, broken home history or delinquency, poor coping skills), their partnership (early parenthood, one-parent family, unwanted pregnancy, marital discord) and the family environment (overcrowding, poor social integration and support, severe chronic life difficulties), was assessed according to an ‘enriched’ family adversity index by a standardized parent interview conducted at each assessment until the age of 11 years (range 0-9, M=2.95; SD=2.05). In this report, a modified version (excluding parental psychiatric diagnoses) of the adversity score was used when compared to that used in a previous publication .

*Life events.* To assess exposure to life stress (LS; 15-25years), a semi-structured parent interview was conducted at the age of 15 years. From the age of 19 years onwards, the young adults were interviewed. The interview, a modified and shortened version of the Munich Events List , evaluated the occurrence of adverse life events during a period of 1 year prior to the assessment. The items (42–59, adjusted for developmental period) covered all relevant areas of children’s and young adults’ LS including family, school, parents, health, legal troubles, and living conditions, such as birth of a sibling, death of a close relative or parents` separation. A composite score was computed by summing up the z-standardized scores from the assessments.

## Reward task

An anticipation cue is presented, followed by the flash target which is followed by feedback about their current balance. The cues consistently predicted different types of reward anticipation: either a smiley signaling that responding sufficiently fast after the flash target would yield monetary feedback (“0.50 Euro” or “0.00 Euro"), or a scrambled smiley indicating verbal feedback (“Fast reaction!” or “Not fast enough!”). Boost trials with a monetary reward of 2 Euro instead of 0.50 Euro occurred after approximately every eighth win trial, in order to improve the participants’ level of motivation. In total, 50 monetary and 50 verbal feedback trials were presented in a pseudo-randomized order. The reaction time (RT) window (common to both reward conditions, with a maximum of 1 sec.) was adaptively tailored to the individual RTs to yield comparable winnings across participants (~60%). After every trial, the participants were informed about their current account balance. The time window between the beginning of cue presentation and feedback was jittered mainly by varying cue duration (3-5sec).

## fMRI parameters and data analysis

The imaging protocol consisted of a localizer scan followed by a BOLD-sensitive T2*-weighted echoplanar imaging sequence and a structural T1-weighted sequence. For functional imaging, a total of 400 volumes with 36 slices (matrix 64x64, resolution 3.43x3.43x3 mm, repetition time=2210ms, echo time=28 ms, flip angle=90°) covering the whole brain were acquired. The slices were inclined 20° from the anterior/posterior commissure level to minimize dropout artifacts in orbitofrontal and mediotemporal regions. The first 4 fMRI volumes were discarded to allow longitudinal magnetization to reach equilibrium. Preprocessing included slice time correction of the volumes to the first slice, realignment to correct for movement artifacts, coregistration of functional and anatomical data, spatial normalization to standard MNI (Montreal Neurological Institute) space and smoothing with a Gaussian kernel of 8mm full-width-at-half-maximum (FWHM). In the first level analysis eight regressors of interest (laughing and scrambled smiley, flash, response, monetary and verbal, win and no-win trials, respectively) were created and convolved with the SPM hemodynamic response function (HRF). A further six movement parameters were included as regressors of no interest. The analysis of Regions of Significance (RoS) provides a test of the statistical significance of the association between a moderator (i.e., familial risk) and an outcome variable at all values of an independent variable (i.e., early maternal care) that fall within a range of interest. It allows for marking the specific values of the independent variable below which and above which the regression lines for the two studied groups differ significantly (regions marked in grey).

# Results

## Task effects

Robust caudate and putamen activation emerged during the anticipation of reward (caudate right: t(171)=19.80, p_FWE_<.001; left: t(171)=18.98, p_FWE_<.001; putamen right: t(171)=19.83, p_FWE_<.001; left: t(171)= 19.84, p_FWE_<.001) as well as during the delivery (caudate right: t(171)=10.99, p_FWE_<.001; left: t(171)=10.19, p_FWE_<.001; putamen right: t(171)=12.52, p_FWE_<.001; left: t(171)=12.24, p_FWE_<.001).

## Interaction effects

Whole-brain results during anticipation are depicted in Table S1. With regard to reward anticipation, the interaction remained significant when performed according to the recommendations by Keller (*t*(162)=3.56, p_FWE_=.006). During reward delivery, the interaction fell short of significance when all these interactions were included (*t*(162)=2.72, p_FWE_=.06). A break-down of the pooled delivery contrast in won versus not won separately for the monetary and the verbal condition, yielded only significant effects in the former (t(166)=3.15, p_FWE_=.02), with, however, results pointing in the same direction during the verbal condition, albeit not significant after FWE correction.

## Interaction effects on child and adolescent psychiatry

The interaction with regard to ADHD remained significant when all covariate by predictor interactions were included (β=-.38, p=.007). No interaction effect was found with regard to internalizing diagnoses (p=.80). Likewise, the interaction between maternal responsive ness and familial risk did not predict the amount of internalizing diagnoses (p=.47).

## Interaction effects on psychopathology during later life

An interaction effect between maternal stimulation and familial liability emerged with respect to aggression (β=-1.15, p=.018) and externalizing behavior during later life (β=-.96, p=.04). The findings indicate lower levels of aggression (β=-.75, p=.04)/ externalizing behavior (β=-.68, p=.058) with increasing stimulation in individuals with familial liability, while no significant effects were found in the low-risk group. This underlines that the effects persisted into adulthood.

When performed according to Keller , the effects remained significant with regard to aggression (β=-1.06, p=.03), however, fell short of significance considering externalizing behavior (p=.057).

## Specificity of the effects

Given that environmental adversity has previously been related to differential reward processing , an additional control for psychosocial adversity did not change the results (caudate responding during anticipation: *t*(165)=3.66, p_FWE_=.004; delivery: *t*(165)=2.88, p_FWE_=.04). This was also true when adjusting for life events occurring after the age of 11 years until the 25-year assessment (caudate responding during anticipation: t(165)=3.59, p_FWE_=.005; delivery: t(165)=2.92, p_FWE_=.04).

Likewise, the results remained significant after control for lifetime internalizing psychopathology in the offspring (anticipation: *t*(165)=3.63, p_FWE_=.005; delivery: *t*(165)=2.94, p_FWE_=.04; ADHD: β=-.38, p=.005). The same applied when parental affective disorders were excluded from the familial risk score (anticipation: *t*(166)=2.92, p_FWE_=.03; delivery: *t*(166)=3.48, p_FWE_=.008; ADHD: β=-.31, p=.02).

The interactions with regard to caudate activity during reward anticipation [*t*(159)=3.74, p_FWE_=.003] and ADHD (β=-.42, p=.008) were similar when adjusted for all covariate (including environmental adversity) by predictor interactions and but fall short of significance during delivery [*t*(159)=2.49, p_FWE_=.10]. When adjusting for internalizing diagnoses in the offspring, the pattern looked similar when all predictor by covariate interactions were included (caudate activity during anticipation: *t*(160)=4.12, p_FWE_=.001; caudate activity during delivery: *t*(160)=2.57, p_FWE_=.08; ADHD: β=-.39, p=.006).

Given the nature of the caretaker analyses, analyses were rerun when considering maternal (N=64) and paternal psychopathology (N=43) separately. Interestingly, while the interaction between paternal diagnoses and maternal stimulation was not significant with regard to all outcomes (anticipation and delivery: p_FWE_>.05; ADHD: p=.11), the interaction between maternal diagnoses and maternal stimulation was related to the amount of ADHD diagnoses (β=-.28, p=.04) and caudate activity during anticipation (*t*(166)=4.04, p_FWE_=.001) with a trend for significance during delivery (*t*(166)=2.65, p_FWE_=.07).

# Discussion

Generally speaking, familial risk may represent both genetic and environmental influences. In line with a confounding of genetic and environmental transmission effects, a gene-environment correlation was found between high familial risk and decreased maternal stimulation. By controlling for psychosocial adversity and stressful life events, we ensured that the observed interaction patterns could not be attributed to a general high level of environmental stress. Hence, it is intriguing to assume that the presence of parental psychiatric disorder might confer a genetic rather than an environmental risk.

References

Basten, M. M., Althoff, R. R., Tiemeier, H., Jaddoe, V. W., Hofman, A., Hudziak, J. J.*, et al.* (2013). The dysregulation profile in young children: empirically defined classes in the Generation R study. *Journal of the American Academy of Child and Adolescent Psychiatry*, **52**(8), 841-850 e842.

Boecker-Schlier, R., Holz, N. E., Hohm, E., Zohsel, K., Blomeyer, D., Buchmann, A. F.*, et al.* (2017). Association between pubertal stage at first drink and neural reward processing in early adulthood. *Addiction Biology*, **22**(5), 1402-1415.

Boecker, R., Holz, N. E., Buchmann, A. F., Blomeyer, D., Plichta, M. M., Wolf, I.*, et al.* (2014). Impact of early life adversity on reward processing in young adults: EEG-fMRI results from a prospective study over 25 years. *PloS One*, **9**(8), e104185.

Breaux, R. P., Harvey, E. A. & Lugo-Candelas, C. I. (2014). The role of parent psychopathology in the development of preschool children with behavior problems. *Journal of Clinical Child and Adolescent Psychology*, **43**(5), 777-790.

Brennan, P. A., Hammen, C., Katz, A. R. & Le Brocque, R. M. (2002). Maternal depression, paternal psychopathology, and adolescent diagnostic outcomes. *Journal of Consulting and Clinical Psychology*, **70**(5), 1075-1085.

Byrd, A. L., Loeber, R. & Pardini, D. A. (2014). Antisocial behavior, psychopathic features and abnormalities in reward and punishment processing in youth. *Clinical Child and Family Psychology Review*, **17**(2), 125-156.

Forbes, E. E. (2011). fMRI studies of reward processing in adolescent depression. *Neuropsychopharmacology*, **36**(1), 372-373.

Hicks, B. M., Foster, K. T., Iacono, W. G. & McGue, M. (2013). Genetic and environmental influences on the familial transmission of externalizing disorders in adoptive and twin offspring. *JAMA Psychiatry*, **70**(10), 1076-1083.

Holz, N. E., Boecker-Schlier, R., Buchmann, A. F., Blomeyer, D., Jennen-Steinmetz, C., Baumeister, S.*, et al.* (2017). Ventral striatum and amygdala activity as convergence sites for early adversity and conduct disorder. *Social Cognitive and Affective Neuroscience*, **12**(2), 261-272.

Hussong, A. M., Huang, W., Curran, P. J., Chassin, L. & Zucker, R. A. (2010). Parent alcoholism impacts the severity and timing of children's externalizing symptoms. *Journal of Abnormal Child Psychology*, **38**(3), 367-380.

Keller, M. C. (2014). Gene x environment interaction studies have not properly controlled for potential confounders: the problem and the (simple) solution. *Biological Psychiatry*, **75**(1), 18-24.

Laucht, M., Esser, G., Baving, L., Gerhold, M., Hoesch, I., Ihle, W.*, et al.* (2000). Behavioral sequelae of perinatal insults and early family adversity at 8 years of age. *Journal of the American Academy of Child and Adolescent Psychiatry*, **39**(10), 1229-1237.

Laucht, M., Esser, G. & Schmidt, M. H. (1997). Developmental outcome of infants born with biological and psychosocial risks. *Journal of Child Psychology and Psychiatry*, **38**(7), 843-853.

Maier-Diewald, W., Wittchen, H.-U., Hecht, H. & Werner-Eilert, K. (1983). *[Munich Interview for the Assessment of Life Events and Conditions - Manual]*. Munich: Max Planck Institute of Psychiatry.

Marmorstein, N. R., Iacono, W. G. & McGue, M. (2009). Alcohol and illicit drug dependence among parents: associations with offspring externalizing disorders. *Psychological Medicine*, **39**(1), 149-155.

Nigg, J. T. & Hinshaw, S. P. (1998). Parent personality traits and psychopathology associated with antisocial behaviors in childhood attention-deficit hyperactivity disorder. *Journal of Child Psychology and Psychiatry and Allied Disciplines*, **39**(2), 145-159.

Renk, K., Oliveros, A., Roddenberry, A., Klein, J., Sieger, K., Roberts, R.*, et al.* (2007). The relationship between maternal and paternal psychological symptoms and ratings of adolescent functioning. *Journal of Adolescence*, **30**(3), 467-485.

Richey, J. A., Rittenberg, A., Hughes, L., Damiano, C. R., Sabatino, A., Miller, S.*, et al.* (2014). Common and distinct neural features of social and non-social reward processing in autism and social anxiety disorder. *Social Cognitive and Affective Neuroscience*, **9**(3), 367-377.

Stepp, S. D., Whalen, D. J., Pilkonis, P. A., Hipwell, A. E. & Levine, M. D. (2012). Children of mothers with borderline personality disorder: identifying parenting behaviors as potential targets for intervention. *Personality Disorders*, **3**(1), 76-91.

Wittchen, H. U., Zaudig, M. & Fydrich, T. (1997). *SKID - Strukturiertes Klinisches Interview DSM-IV Achse I und II.* Göttingen (DE): Hogrefe.

***Tables***

**Table S1. Negative association with familial risk during reward anticipation at p=.001 uncorrected.**

|  |  |  |  |  | **MNI coordinates** | | |
| --- | --- | --- | --- | --- | --- | --- | --- |
| **Region** | **Cluster size** | **p(FWE-corr)** | **T-value** | **p(unc)** | **x** | **y** | **z** |
| Anterior Cingulate | 4271 | 0.045^a^ | 4.61 | <.001 | 18 | 34 | 16 |
| Medial Frontal Gyrus |  | 0.063 | 4.52 | <.001 | -16 | 42 | 24 |
| Caudate |  | 0.113 | 4.35 | <.001 | 16 | 10 | 20 |
| Lingual Gyrus | 228 | 0.053 | 4.57 | <.001 | 28 | -82 | 2 |
| Fusiform Gyrus |  | 0.791 | 3.52 | <.001 | 32 | -72 | -4 |
| Lingual Gyrus |  | 0.953 | 3.26 | .001 | 24 | -70 | 4 |
| Superior Temporal Gyrus | 75 | 0.136 | 4.29 | <.001 | 68 | -38 | 20 |
| Cuneus | 1747 | 0.165 | 4.22 | <.001 | 22 | -78 | 26 |
| Cuneus |  | 0.213 | 4.14 | <.001 | -4 | -72 | 24 |
|  |  | 0.241 | 4.10 | <.001 | -22 | -54 | 24 |
| Caudate | 215 | 0.173 | 4.21 | <.001 | 42 | -42 | 6 |
| Hippocampus |  | 0.699 | 3.61 | <.001 | 32 | -28 | -16 |
| Parahippocampal Gyrus |  | 0.706 | 3.60 | <.001 | 42 | -38 | -10 |
|  | 274 | 0.224 | 4.12 | <.001 | -42 | -46 | 8 |
| Middle Temporal Gyrus |  | 0.425 | 3.88 | <.001 | -42 | -54 | 12 |
| Middle Temporal Gyrus |  | 0.722 | 3.59 | <.001 | -46 | -58 | 6 |
| Superior Temporal Gyrus | 115 | 0.273 | 4.05 | <.001 | -48 | -22 | 6 |
| Transverse Temporal Gyrus |  | 0.808 | 3.50 | <.001 | -54 | -14 | 10 |
| Cingulate Gyrus | 130 | 0.275 | 4.05 | <.001 | 28 | -40 | 44 |
| Cingulate Gyrus |  | 0.892 | 3.38 | <.001 | 20 | -34 | 40 |
| Postcentral Gyrus | 360 | 0.277 | 4.05 | <.001 | 34 | -18 | 40 |
| Postcentral Gyrus |  | 0.371 | 3.93 | <.001 | 48 | -24 | 56 |
| Postcentral Gyrus |  | 0.877 | 3.41 | <.001 | 40 | -28 | 68 |
| Sub-Gyral | 39 | 0.302 | 4.01 | <.001 | -48 | -20 | -18 |
| Declive | 273 | 0.418 | 3.88 | <.001 | -38 | -66 | -20 |
| Inferior Occipital Gyrus |  | 0.823 | 3.48 | <.001 | -48 | -74 | 0 |
| Inferior Occipital Gyrus |  | 0.942 | 3.28 | .001 | -40 | -72 | -2 |
| * | 280 | 0.483 | 3.82 | <.001 | 10 | -52 | -28 |
| Cerebellar Tonsil |  | 0.788 | 3.52 | <.001 | 24 | -66 | -36 |
| Inferior Semi-Lunar Lobule |  | 0.796 | 3.51 | <.001 | 30 | -72 | -46 |
| Postcentral Gyrus | 137 | 0.513 | 3.79 | <.001 | -8 | -48 | 72 |
| Precentral Gyrus | 40 | 0.524 | 3.78 | <.001 | -44 | -2 | 46 |
| Superior Parietal Lobule | 28 | 0.526 | 3.78 | <.001 | 34 | -52 | 68 |
| Thalamus | 79 | 0.542 | 3.76 | <.001 | -26 | -26 | 4 |
| Hippocampus | 11 | 0.585 | 3.72 | <.001 | -32 | -26 | -14 |
| Postcentral Gyrus | 85 | 0.613 | 3.69 | <.001 | 66 | -16 | 26 |
| Precentral Gyrus |  | 0.746 | 3.56 | <.001 | 64 | -14 | 34 |
| Cingulate Gyrus | 75 | 0.639 | 3.67 | <.001 | -12 | -26 | 38 |
| Cingulate Gyrus |  | 0.744 | 3.57 | <.001 | -14 | -18 | 38 |
| Thalamus | 174 | 0.653 | 3.66 | <.001 | -4 | -14 | 4 |
| Thalamus |  | 0.715 | 3.59 | <.001 | 2 | -24 | 6 |
| Thalamus |  | 0.849 | 3.44 | <.001 | -14 | -18 | 2 |
| Superior Frontal Gyrus | 34 | 0.698 | 3.61 | <.001 | -20 | 16 | 60 |
| Inferior Occipital Gyrus | 67 | 0.717 | 3.59 | <.001 | -8 | -96 | -4 |
| Middle Frontal Gyrus | 16 | 0.728 | 3.58 | <.001 | 34 | 44 | 38 |
| Posterior Cingulate | 45 | 0.742 | 3.57 | <.001 | 6 | -58 | 12 |
| Thalamus | 31 | 0.747 | 3.56 | <.001 | 26 | -34 | 2 |
| Cerebellar Tonsil | 30 | 0.754 | 3.56 | <.001 | -10 | -48 | -48 |
| * | 11 | 0.784 | 3.52 | <.001 | -10 | -42 | -28 |
| Medial Frontal Gyrus | 8 | 0.825 | 3.47 | <.001 | 18 | 60 | -4 |
| Precentral Gyrus | 13 | 0.828 | 3.47 | <.001 | 46 | -2 | 28 |
|  | 18 | 0.832 | 3.47 | <.001 | 38 | -72 | 12 |
| Culmen | 11 | 0.838 | 3.46 | <.001 | 26 | -52 | -10 |
| Pyramis | 30 | 0.841 | 3.46 | <.001 | 6 | -78 | -24 |
| Postcentral Gyrus | 34 | 0.854 | 3.44 | <.001 | 54 | -14 | 26 |
| Postcentral Gyrus |  | 0.975 | 3.18 | .001 | 48 | -20 | 30 |
| Middle Frontal Gyrus | 9 | 0.896 | 3.38 | <.001 | 46 | 4 | 56 |
| Paracentral Lobule | 45 | 0.915 | 3.34 | .001 | 6 | -34 | 74 |
| Paracentral Lobule |  | 0.918 | 3.34 | .001 | 10 | -30 | 80 |
| Precentral Gyrus | 18 | 0.931 | 3.31 | .001 | 60 | 10 | 30 |
| Precentral Gyrus |  | 0.939 | 3.29 | .001 | 56 | 6 | 42 |
| Cerebellar Tonsil | 19 | 0.934 | 3.30 | .001 | 38 | -58 | -46 |
| Claustrum | 7 | 0.934 | 3.30 | .001 | 34 | -22 | 6 |
| Thalamus | 5 | 0.937 | 3.30 | .001 | -4 | -30 | -2 |
| Paracentral Lobule | 7 | 0.937 | 3.30 | .001 | 14 | -36 | 70 |
| Postcentral Gyrus | 8 | 0.941 | 3.29 | .001 | -46 | -22 | 34 |
| Fusiform Gyrus | 8 | 0.942 | 3.29 | .001 | 44 | -68 | -6 |
| Superior Temporal Gyrus | 8 | 0.946 | 3.28 | .001 | 52 | -30 | -10 |
| Middle Temporal Gyrus |  | 0.972 | 3.19 | .001 | 58 | -26 | -14 |
| Cingulate Gyrus | 9 | 0.947 | 3.27 | .001 | -18 | -42 | 44 |
| Uvula | 21 | 0.951 | 3.26 | .001 | -4 | -78 | -30 |
| Thalamus | 9 | 0.970 | 3.20 | .001 | 4 | -10 | 16 |

Note: ^a^ survives whole-brain FWE correction at p<.05.

**Table S2. Positive association with familial risk during reward delivery at**

|  |  |  |  |  | **MNI coordinates** | | |
| --- | --- | --- | --- | --- | --- | --- | --- |
| **Region** | **Cluster size** | **p (FWE)** | **T-value** | **p (uncorr)** | **x** | **y** | **z** |
| Putamen | 182 | .48 | 3.90 | <.001 | 20 | 10 | 2 |
| Caudate | 191 | .48 | 3.90 | <.001 | -16 | 14 | 0 |
|  |  | .65 | 3.75 | <.001 | -24 | 2 | 10 |
|  |  | .88 | 3.51 | <.001 | -24 | 10 | 6 |
| Middle Frontal Gyrus | 51 | .73 | 3.67 | <.001 | 28 | -70 | -24 |

**p=.001 uncorrected.**

**Table S3. Interaction between familial risk and maternal stimulation with regard to reward anticipation at p=.001 uncorrected.**

|  |  |  |  |  | **MNI coordinates** | | |
| --- | --- | --- | --- | --- | --- | --- | --- |
| **Region** | **Cluster size** | **p(FWE-corr)** | **T-value** | **p(unc)** | **x** | **y** | **z** |
| Suppl. Motor Area | 3690 | 0.009 ^a^ | 5.08 | <.001 | 2 | 8 | 70 |
| Cingulum |  | 0.019 ^a^ | 4.88 | <.001 | 4 | 26 | 40 |
| Middle Frontal Gyrus |  | 0.046 ^a^ | 4.64 | <.001 | -18 | 46 | 26 |
| Middle Frontal Gyrus | 1271 | 0.026 ^a^ | 4.80 | <.001 | 26 | 40 | 30 |
| Middle Frontal Gyrus |  | 0.088 | 4.46 | <.001 | 36 | 54 | 18 |
| Medial Frontal Gyrus |  | 0.095 | 4.44 | <.001 | 16 | 44 | 32 |
| Insula | 891 | 0.040 ^a^ | 4.68 | <.001 | 44 | 6 | 0 |
| Inferior Frontal Gyrus |  | 0.289 | 4.07 | <.001 | 54 | 16 | -4 |
| Inferior Frontal Gyrus |  | 0.327 | 4.02 | <.001 | 44 | 34 | -2 |
| Parahippocampal Gyrus | 215 | 0.101 | 4.42 | <.001 | -34 | -42 | -2 |
| Sub-Gyral |  | 0.709 | 3.64 | <.001 | -38 | -32 | -4 |
| Anterior Cingulate | 157 | 0.114 | 4.38 | <.001 | -18 | 42 | -2 |
| Precentral Gyrus | 664 | 0.265 | 4.10 | <.001 | -52 | 16 | 2 |
| Superior Temporal Gyrus |  | 0.470 | 3.87 | <.001 | -56 | 6 | -2 |
| Insula |  | 0.521 | 3.82 | <.001 | -36 | 2 | 10 |
| Claustrum | 65 | 0.449 | 3.89 | <.001 | 38 | -14 | -10 |
| Inferior Frontal Gyrus | 74 | 0.468 | 3.87 | <.001 | 58 | 22 | 18 |
| Middle Frontal Gyrus |  | 0.705 | 3.65 | <.001 | 52 | 32 | 18 |
| Inferior Parietal Lobule | 167 | 0.593 | 3.75 | <.001 | 64 | -30 | 24 |
| Insula |  | 0.908 | 3.40 | <.001 | 54 | -34 | 28 |
| Postcentral Gyrus |  | 0.979 | 3.21 | <.001 | 66 | -22 | 20 |
| Caudate | 52 | 0.594 | 3.75 | <.001 | 40 | -36 | 6 |
| Caudate |  | 0.807 | 3.54 | <.001 | 42 | -40 | -4 |
| Inferior Frontal Gyrus | 92 | 0.637 | 3.71 | <.001 | -44 | 46 | -2 |
| Middle Frontal Gyrus |  | 0.924 | 3.37 | <.001 | -38 | 52 | 2 |
| Parahippocampal Gyrus | 189 | 0.699 | 3.65 | <.001 | -6 | -40 | 6 |
| Thalamus |  | 0.902 | 3.41 | <.001 | 6 | -32 | 6 |
| Sub-Gyral | 59 | 0.708 | 3.65 | <.001 | -22 | -34 | 58 |
| Tuber | 40 | 0.721 | 3.63 | <.001 | 42 | -70 | -26 |
| Caudate | 118 | 0.722 | 3.63 | <.001 | -12 | 22 | 6 |
| Caudate |  | 0.737 | 3.62 | <.001 | -4 | 22 | 4 |
| Caudate |  | 0.893 | 3.43 | <.001 | -12 | 32 | 6 |
| Superior Temporal Gyrus | 26 | 0.730 | 3.62 | <.001 | -64 | -38 | 24 |
| Declive | 41 | 0.826 | 3.52 | <.001 | 40 | -66 | -8 |
| Parahippocampal Gyrus |  | 0.909 | 3.40 | <.001 | 38 | -58 | -4 |
| Precuneus | 22 | 0.849 | 3.49 | <.001 | 16 | -34 | 58 |
| Inferior Frontal Gyrus | 13 | 0.853 | 3.49 | <.001 | 50 | 42 | 6 |
| Middle Frontal Gyrus | 25 | 0.875 | 3.46 | <.001 | 36 | 6 | 56 |
| Precuneus | 18 | 0.881 | 3.45 | <.001 | 34 | -42 | 56 |
| Caudate | 24 | 0.886 | 3.44 | <.001 | 16 | 12 | 18 |
| Middle Frontal Gyrus | 21 | 0.886 | 3.44 | <.001 | 52 | 12 | 40 |
| Middle Frontal Gyrus | 16 | 0.888 | 3.44 | <.001 | 34 | -4 | 40 |
| Postcentral Gyrus | 14 | 0.915 | 3.39 | <.001 | 26 | -26 | 50 |
| Declive | 14 | 0.921 | 3.38 | <.001 | -36 | -76 | -20 |
| Inferior Parietal Lobule | 50 | 0.929 | 3.36 | <.001 | 46 | -36 | 56 |
| Inferior Parietal Lobule |  | 0.933 | 3.35 | <.001 | 52 | -32 | 50 |
| Thalamus | 11 | 0.939 | 3.34 | <.001 | 18 | -30 | 10 |
| Anterior Cingulate | 16 | 0.940 | 3.34 | <.001 | 4 | 52 | -2 |
| Superior Temporal Gyrus | 5 | 0.941 | 3.34 | <.001 | -52 | -18 | -6 |
| Precentral Gyrus | 9 | 0.941 | 3.34 | <.001 | -58 | -2 | 12 |
| Caudate | 9 | 0.947 | 3.32 | <.001 | 24 | -22 | 28 |
| Posterior Cingulate | 5 | 0.972 | 3.24 | <.001 | -26 | -58 | 22 |
| Middle Frontal Gyrus | 5 | 0.975 | 3.23 | <.001 | 36 | 54 | -6 |
| Tuber | 6 | 0.977 | 3.22 | <.001 | -40 | -64 | -24 |
| Note: ^a^ survives whole-brain FWE correction at p<.05. | | | | | | |  |

**Table S4. Interaction between familial risk and child responsiveness with regard to reward anticipation at p=.001 uncorrected.**

|  |  |  |  |  | **MNI coordinates** | | |
| --- | --- | --- | --- | --- | --- | --- | --- |
| **Region** | **Cluster size** | **p(FWE-corr)** | **T-value** | **p(unc)** | **x** | **y** | **z** |
| Superior Frontal Gyrus | 6 | 0.889 | 3.42 | <.001 | -14 | 54 | 12 |
| Postcentral Gyrus | 6 | 0.936 | 3.34 | .001 | -28 | -34 | 50 |

**Table S5. Interaction between familial risk and maternal responsiveness with regard to reward anticipation at p=.001 uncorrected.**

|  |  |  |  |  | **MNI coordinates** | | |
| --- | --- | --- | --- | --- | --- | --- | --- |
| **Region** | **Cluster size** | **p(FWE-corr)** | **T-value** | **p(unc)** | **x** | **y** | **z** |
| Anterior Cingulate | 54 | 0.233 | 4.14 | <.001 | 14 | 38 | -2 |
| Superior Frontal Gyrus | 70 | 0.554 | 3.79 | <.001 | -10 | 46 | 34 |
| Superior Frontal Gyrus | 73 | 0.780 | 3.57 | <.001 | -14 | 60 | 20 |
| Superior Frontal Gyrus |  | 0.890 | 3.43 | <.001 | 0 | 54 | 22 |
|  |  | 0.909 | 3.39 | <.001 | 14 | 58 | 18 |
| Temporal Pole | 8 | 0.861 | 3.47 | <.001 | 52 | 10 | -24 |

**Table S6. Interaction between familial risk and maternal stimulation with regard to reward delivery at p=.001 uncorrected.**

|  |  |  |  |  | **MNI coordinates** | | |
| --- | --- | --- | --- | --- | --- | --- | --- |
| **Region** | **Cluster size** | **p(FWE-corr)** | **T-value** | **p(unc)** | **x** | **y** | **z** |
| Hippocampus | 16 | 0.80 | 3.59 | <.001 | -32 | -10 | -22 |

**Table S7. Interaction between familial risk and infant responsiveness with regard to reward delivery at p=.001 uncorrected.**

|  |  |  |  |  | **MNI coordinates** | | |
| --- | --- | --- | --- | --- | --- | --- | --- |
| **Region** | **Cluster size** | **p(FWE-corr)** | **T-value** | **p(unc)** | **x** | **y** | **z** |
| Lingual Gyrus | 68 | 0.60 | 3.78 | <.001 | 16 | -52 | -4 |
| Lingual Gyrus | 19 | 0.76 | 3.63 | <.001 | -16 | -44 | 0 |
| Cuneus | 68 | 0.80 | 3.58 | <.001 | 14 | -72 | 30 |
| Lingual Gyrus | 32 | 0.93 | 3.41 | <.001 | -12 | -60 | -6 |

**Table S8. Interaction between familial risk and maternal responsiveness with regard to reward delivery at p=.001 uncorrected.**

|  |  |  |  |  | **MNI coordinates** | | |
| --- | --- | --- | --- | --- | --- | --- | --- |
| **Region** | **Cluster size** | **p(FWE-corr)** | **T-value** | **p(unc)** | **x** | **y** | **z** |
| Lingual Gyrus | 560 | 0.133 | 4.36 | <.001 | -10 | -62 | -4 |
|  |  | 0.233 | 4.18 | <.001 | 10 | -58 | -4 |
|  |  | 0.612 | 3.77 | <.001 | 10 | -66 | -8 |
| Middle Frontal Gyrus | 32 | 0.672 | 3.72 | <.001 | 36 | 60 | 16 |
| Amygdala | 12 | 0.786 | 3.60 | <.001 | 32 | -6 | -24 |
| Superior Occipital Gyrus | 17 | 0.878 | 3.49 | <.001 | -16 | -98 | 22 |
| Fusiform Gyrus | 9 | 0.961 | 3.32 | .001 | -42 | -44 | -12 |
| Superior Parietal Lobule | 9 | 0.969 | 3.30 | .001 | 36 | -42 | 48 |

***Supplementary Figure Legends***

Figure S1. Region of Significance analysis of the interaction between maternal stimulation and familial risk with regard to (A) reward anticipation and (B) reward delivery.

Figure S2. A. Maternal stimulation (MS) x familial risk interaction on supplementary motor area activity during reward anticipation.

Figure S3. Region of Significance analysis of the interaction between maternal stimulation and familial risk with regard to ADHD.
